# Surgeon Training in Telerobotic Surgery via a Hardware-in-the-Loop Simulator

**DOI:** 10.1155/2017/6702919

**Published:** 2017-08-03

**Authors:** Xiao Li, Homa Alemzadeh, Daniel Chen, Zbigniew Kalbarczyk, Ravishankar K. Iyer, Thenkurussi Kesavadas

**Affiliations:** ^1^Department of Mechanical Engineering, University of Illinois, Urbana, IL 61801, USA; ^2^Department of Electrical and Computer Engineering, University of Virginia, Charlottesville, VA 22903, USA; ^3^Department of Electrical and Computer Engineering, University of Illinois, Urbana, IL 61801, USA; ^4^Department of Industrial and Enterprise Systems Engineering, University of Illinois, Urbana, IL 61801, USA

## Abstract

This work presents a software and hardware framework for a telerobotic surgery safety and motor skill training simulator. The aims are at providing trainees a comprehensive simulator for acquiring essential skills to perform telerobotic surgery. Existing commercial robotic surgery simulators lack features for safety training and optimal motion planning, which are critical factors in ensuring patient safety and efficiency in operation. In this work, we propose a hardware-in-the-loop simulator directly introducing these two features. The proposed simulator is built upon the Raven-II™ open source surgical robot, integrated with a physics engine and a safety hazard injection engine. Also, a Fast Marching Tree-based motion planning algorithm is used to help trainee learn the optimal instrument motion patterns. The main contributions of this work are (1) reproducing safety hazards events, related to da Vinci™ system, reported to the FDA MAUDE database, with a novel haptic feedback strategy to provide feedback to the operator when the underlying dynamics differ from the real robot's states so that the operator will be aware and can mitigate the negative impact of the safety-critical events, and (2) using motion planner to generate semioptimal path in an interactive robotic surgery training environment.

## 1. Introduction

**Figure 1:**
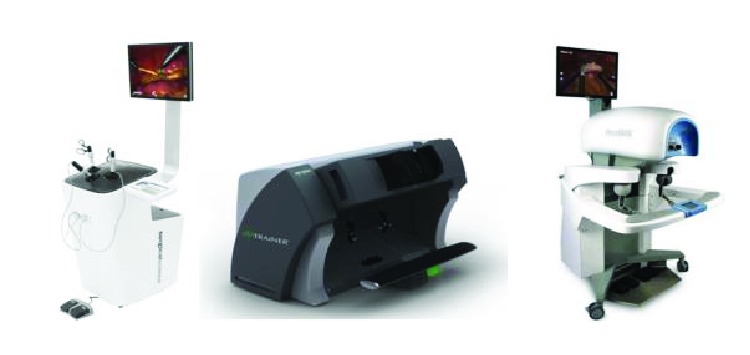
From left to right: LapSim haptic system, dV-trainer, and RoSS.

The field of surgical robotics has been rapidly expanding over the last decade [1]. Robot-assisted surgery is the preferred technique for a variety of minimally invasive procedures worldwide. Simulation-based learning and training is now a standard in robotic surgery because healthcare professionals improve performance and reduce errors through comprehensive medical care simulation [2, 3]. Simulation can bridge the gap in learning robotic surgery skills without accidentally harming the patient. For example, LapSim Haptic System™ is a laparoscopic surgery simulator with realistic hardware interface and tactile feedback, which is mainly used for near-field nonteleoperated surgery training [4]. Other commercial surgical simulators on the market such as Mimic's dV-Trainer™ [5] and Simulated Surgical System's RoSS [6] provide basic motor skills training modules using virtual reality with surgeon console similar to the da Vinci surgical system, to provide life-like simulation and help prepare surgeons.  Figure 1 shows these simulators' profiles. A key issue with simulation-based surgical training is the lack of safety-critical incident scenarios in simulation-based curricula, which is critical in bringing this form of surgical education to practice. Current surgical simulators focus on better graphics rendering and curriculum of goal achievement (success in arriving at goals while ignoring intermediate motion patterns) rather than real robot dynamics motion as well as teaching the surgeon optimal motion pattern and path in an obstacle-surrounded environment.

Our previous study of adverse events reported to the U.S. Food and Drug Administration (FDA) Manufacturer and User Facility Device Experience (MAUDE) database showed that despite significant improvements in robotic surgery technology through the years and broader adoption of the robotic approach, there are ongoing occurrences of safety incidents that negatively impact patients. The number of injury and death events per procedure has stayed relatively constant since 2007, with an average of 83.4 events per 100,000 procedures [7]. Although, these incidents are often caused by accidental malfunctions or technical problems with the robot and steep learning curves, it has also been shown that surgical robots can be subject to malicious cyber-attacks that impact patient safety and progress of surgery [8, 9]. The ability of current robotic surgery technology to automatically mitigate the impact of safety incidents still lags other safety-critical industries, such as commercial aviation. In such industries, great effort has been spent over the years on improving safety practices by providing comprehensive simulation-based training that includes operation in the presence of safety-critical failures [10]. In current robotic surgeon training, the emphasis is on improving surgical skills and not on handling safety-critical events and responding to technical problems. Adverse events or accidental machine failures are rarely used as potential scenarios for safety training of surgical teams.

In this work, we are motivated by the idea of simulating safety hazards [11, 12] during robotic surgery training in order to prepare surgeons for handling safety-critical events. The objective is to develop a hardware-in-the-loop simulator platform that emulates realistic safety hazard scenarios in a virtual environment and provides awareness of the impeding hazards to the operator through haptic force feedback. In this work, we use Raven-II [13] surgical robot as the hardware that the operator will be trained with. Previous studies have shown that users trained on the Raven platform can transfer their skills to da Vinci system [14]. We developed a robot-environment interaction model using a physics engine as the robot's nominal state estimator (fault-free run), which runs simultaneously with the Raven-II robot hardware. We also developed a safety hazard injection engine that intentionally and artificially creates adverse events by inserting faults into the robot control system using Software-implemented Fault Injection (SWIFI) [15]. The faults are injected to the control software after the system's automatic safety checks are performed to increase the chance that they cause safety hazards.

The main goal of SWIFI is to validate the effectiveness of fault-tolerance mechanisms by studying system behavior in the presence of simulated faults. Here, we use software-based fault injection techniques to emulate the safety hazards and enable evaluation of human operator performance and response to safety hazards during simulation-based training. Fault injection and cyber-attacks on the safety-critical systems, such as smart grid [16–18], automotive embedded systems [19], and robotic vehicles [20, 21], have been the subject of many studies. They presented attack scenarios that directly target the physical system, the control commands sent to the physical system, or the sensor measurements received from the physical layer to corrupt the state of controller in the cyber-domain (false data injection attacks). In this work, we use fault injection and target the robot control system to corrupt the control commands in a legitimate manner that is not detectable by the robot's safety mechanisms. In our previous work, we showed that these injections could lead to unexpected and sudden jumps of the robotic arms and negatively impact the robot operation and patient safety in just a couple of milliseconds, making it difficult for both automated mechanisms and human operators to respond in a timely manner. Thus, the detection and response mechanisms in real-time surgical cyber-physical systems should be optimized and deployed in such a way that can mitigate the impact of faulty and malicious commands before they even execute in the physical layer [8].

In [22], authors demonstrated content modification attacks on a bilateral teleoperation system and used Lyapunov-based analysis to conclude that if the sent velocity does not equal to the position's derivative, then there is a static attack (linear modification of the states using time-invariant gains). Their method however might suffer from sensitivity to model accuracy, since all analysis is based on model-based Lyapunov analysis. Providing the user with haptic cues using the haptic force calculated based on the difference between desired and actual position of end-effectors in the slave robot was proposed in [23]. Teleoperation for Raven robot uses Interoperable Telerobotics Protocol [24]—sending incremental rather than absolute motion command from master to slave and a human operator is in the control loop to correct the position errors. In transient phase, there is tracking error between the actual slave robot end-effector position and the desired position; therefore, comparing the two to generate the haptic feedback cannot be done accurately. We address this issue by developing a dynamical model for the physical robot (a virtual robot) as the underlying state monitor. The model and robot receive the same motion command from the master, and we use their state difference to create the haptic feedback.

When training a novice surgeon, he/she can acquire some sense of optimality by observing or sensing (through haptics) the robot's execution of an automated task. The motion can be planned optimally by minimizing certain cost functions. Related work in this area focuses on automating some of the real surgery scenarios in different robot-assisted surgery types. Weede et al. in [25] introduced an autonomous camera system including a prediction of interventions, to provide a long-term prediction of the steps a surgeon will perform in the next few minutes and move the endoscope to an optimal position. Combined with vision techniques, automatic positioning and autonomous retrieval of surgical instruments have been achieved in [26, 27]. Chow et al. in [28] showed that vision-guided autonomous knot-tying in robotic-assisted surgery has the potential to be faster than human performance. Kehoe et al. in [29] demonstrated the first reliable autonomous robot performance of surgical subtask, that is, removing tissue fragments using Raven, by generating centralized motion plans through 3D sensing and *trajopt*, a low-level motion planning algorithm based on sequential convex optimization to plan locally optimal, collision-free trajectories simultaneously for both arms. Hu et al. in [30] investigated path planning and semiautomated motion for the scenario of robotic ablation of tumor residues in various shapes using Raven robot, and different metrics were delivered to the surgeon to select candidate path plan. In nonlaparoscopic type of robotic surgery, for example, in needle steering community, efforts have been made in surgical preplanning for the needle type surgical robot [31, 32] and demonstrated in simulation environment [33].

In this work, we present a comprehensive software framework for the telerobotic surgical simulator. The simulator includes a failure scenario generation module which simulates failures during a surgery through fault injections. These failure scenarios can train surgeons to recognize adverse events during a surgery through haptic cues. The optimal trajectory generated by Fast Marching Tree (FMT^∗^) algorithm designed for Raven-II platform at an interactive rate will also help trainee gain an optimal sense of manipulating the surgical instrument.

## 2. Materials and Methods

### 2.1. Simulator Framework

**Figure 2:**
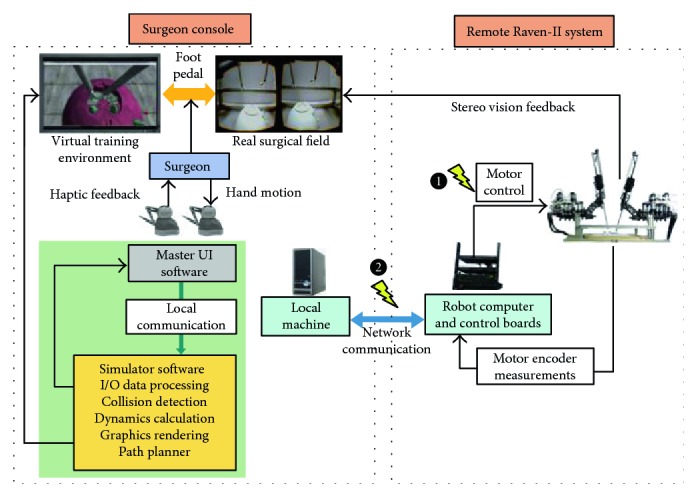
Hardware-in-the-loop surgical simulator architecture.

We design the simulator system based on the Raven-II surgical robot, an open source platform running on top of Robot Operating System (ROS). To develop a surgical simulator with high fidelity in reproducing adverse events, we include the robot hardware in the simulator's execution loop and integrate it with a safety hazard injection engine [8] and a physics engine to simulate the robot dynamics and interaction with environment. The simulator system architecture is shown in Figure 2. Raven-II is a teleoperated surgical robot which uses network communication between the local machine on surgeon's console side and the remote Raven computer. The simulator runs on the local machine and performs dynamics and collision calculations. Two Phantom Omni devices receive the incremental motion command from the operator and then send the data to both the local machine and the remote machine through UDP/IP. A virtual Raven robot and 3D training environment are displayed on the screen of surgeon's console. The graphics are rendered through C++ OpenGL pipeline with a frequency of 30 Hz, while other calculations, for example, haptic loop and physics engine and network data transmission, using multiple threads are being synchronized and run at 1000 Hz, which is the same as the running frequency of Raven's control loop.

The connection between the Surgeon Console and the Raven-II system is bilateral network communication in our hardware-in-the-loop simulator shown in Figure 2. One direction is for transmitting Omni command data from the local machine to the remote Raven system, while the other direction is for sending the robot state data (joint positions and velocities) back to the local machine using TCP/IP socket connection for reliability and to make comparison with the dynamics calculation results in physics engine thread. The haptic force feedback is provided to the operator if the virtual and real Raven's end-effector trajectories do not match (above a predefined threshold). Since perfect transparency (master device force/torque matching the slave's end-effector force/torque) is not possible and is especially challenging for teleoperators with significant nonlinear dynamics and no force sensors mounted at the robot end-effector, we utilize haptics feature for safety propose, rather than for surgical palpation. Because the haptic device sensor/actuator asymmetries can cause instability and robustness issues, we apply a spring-damper model with appropriate gains and saturations for feedback force calculation.

To simulate the safety hazards in real surgery, we integrate the robot control software with a safety hazard injection engine that strategically inserts faults into the control software at critical junctures during operation [8]. More explicitly, the injected faults corrupt either the Omni commands or the motor control commands sent to the Raven hardware after the safety checks are done in the Raven software in robot computer, which is indicated in Figure 2. As a result, unexpected robot motion will generate trajectory errors compared with the underlying model dynamics. Then, we show how the operator can gain awareness of the erroneous robot trajectory in the presence of faults through haptic force feedback.

Beyond the capability of basic motor skills training and simulating adverse events, there are additional features in our simulator that can help improve the surgeon's performance in real surgery. For example, in some scenarios, it is preferable to let the surgeon do a virtual trial, rather than manipulating the actual robot all the time. One of the important capabilities of our simulator is to allow the user to disengage from the actual robot to do a trial movement in the simulator's virtual environment and see the outcomes of virtual motion. If the outcome is satisfactory, then the actual robot could be reengaged to track the recorded command trajectory data and move in an autonomous fashion. The user can also use the path planner to specify the target configuration of the robot arm, and then it will automatically generate trajectory waypoints for the robot arm to track. A foot pedal is placed on the surgeon's side to enable switching between the robot teleoperation mode and pure simulation mode (being disengaged from the robot hardware) and also toggling the view between the real surgical field and virtual environment.

### 2.2. Robot Dynamics Modeling and Training Scenario

In our previous work, we simulated dynamics, numerically integrating the equations of motion derived by Euler-Lagrange (E-L) approach in [34]. This approach provides very little freedom for simulating interactions with the environment. In this work, we integrate a physics engine—Open Dynamics Engine (ODE) into the simulator, to simulate dynamic behaviors of the robot and interactions between the robot manipulators and the environment. ODE is an open source, high-performance library, which relies on a Linear Complementary Problem Solver (LCP solver) [35]. In robotics simulation, ODE is being widely used for a variety of applications [36, 37].

In [34], we obtained the equations of motion by E-L and determined the mechanical properties of the links through CAD models. In ODE setup, we directly specify each link's mesh properties and joint properties, so that we can achieve the same robot motion as in [34] (with no collisions). Besides, ODE has the capability of doing collision checking between primitive objects or meshes and using contact friction models to apply contact forces, which gives the possibility of simulating interactions.

For the training scenario, we use the training model which is widely used in the Fundamentals of Robotic Surgery (FRS) organization, the definitive robotic surgical skills education, training, and validated assessment program [38]. The objectives of using the training model can be found in [38]. Specifically, in our simulator prototype, we made a small modification to the original model (extruded cut half of the top lid) so that the Raven robot arm can be inserted into the cavity to perform the tasks, that is, picking up a cube ring initially placed inside the cavity. The operator will manipulate the robot arms to get used to 3D teleoperation by picking up a ring, transferring it from one arm to the other, and navigating the ring along the loops. The abovementioned motor skills training scenarios can be done in a semiautonomous fashion using the Fast Marching Tree- (FMT^∗^-) based path planner (if only involved one arm motion, dual arm motion case can be split into one arm motion followed by another).

### 2.3. FMT^∗^-Based Path Planner

The path planner utilizes FMT^∗^ algorithm, in which “^∗^” indicates optimality to given cost criteria. An on-off foot pedal is used to activate the path planning functionality. In planning mode, the user is required to use the master device controlling the slave robot in the simulation environment to reach a target configuration. In this phase, collision detection is disabled and just pure kinematic motion is performed. Once the path planner got the goal configuration, it will start to compute dynamically feasible trajectory and execute the motion plan for the arm at interactive rates (only a few seconds for planning and executing motion plans, resp.).

**Figure 3:**
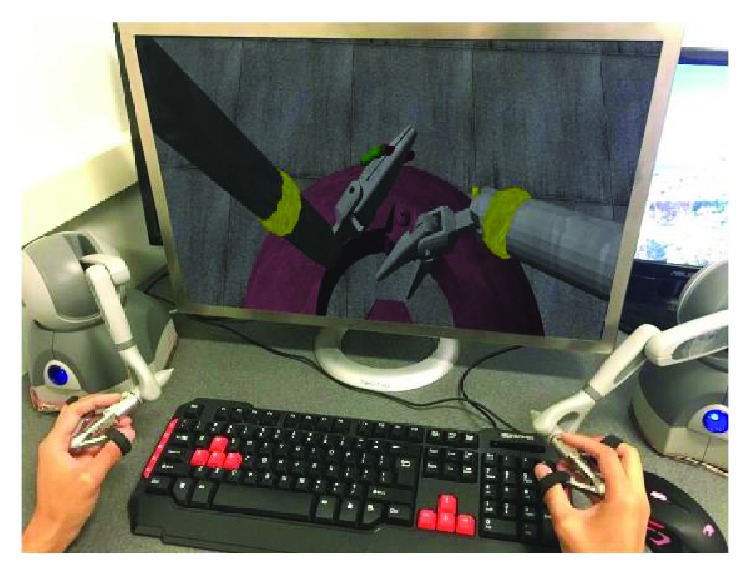
Simulated surgeon console and FRS training model—yellow sphere on each arm indicates the fixed remote motion center, and the cavity of the dome represents a human abdomen area; more details of the model can be found in Figure 7 and Figure 8.

The cavity in the FRS training model is used to simulate the human abdomen (Figure 3), where the inside volume is very constraint and, thus, requires very fine motions of the robot end-effectors. It is preferable to make the surgical instruments (robot arms) automatically inserted or retrieved before or after the surgical procedure. Raven-II robot has two instrument arms that are independent of each other in terms of assembly and controls, and each has seven degrees of freedom (DOF) and six rotational joints plus one translational joint. In high-dimensional space, sampling-based path planning algorithms can explore the configuration space effectively by sampling the collision-free configurations according to a probability distribution (in this work, uniform sampling in feasible joint space is used). Rapidly exploring random tree (RRT) and probabilistic roadmap method (PRM) and their variants have become prevalent in robot path planning applications and literature over the past ten years, especially when the RRT^∗^ and PRM^∗^'s optimality proofs was formally given in [39]. In [39], it is shown that PRM^∗^ and RRT^∗^ are provably asymptotically optimal, that is, the cost of the returned solution converges almost surely to the optimum. However, building the RRT tree or connecting PRM edges require extensive collision checking. In our case, collision checking between triangle meshes will severely hurt the performance. Recently, a new probabilistic sampling-based planning algorithm called Fast Marching Tree (FMT^∗^) was introduced [40]. The algorithm is specifically aimed at solving complex motion planning problems in high-dimensional configuration spaces. This algorithm is proven to be asymptotically optimal and is shown to converge to an optimal solution faster than its state-of-the-art counterparts, namely, PRM^∗^ and RRT^∗^. However, the sacrifice is that it lazily skips collision checks when evaluating the local connections. This lazy collision checking strategy may introduce suboptimal connections, but the crucial property of FMT^∗^ is that such suboptimal connections become vanishingly rare as the number of samples goes to infinity. In both low- and high-dimensional benchmark problems tested in [40], which across a variety of problem instances, ranging in obstacle clutter and in dimensions from 2D to 7D, it is shown that FMT^∗^ outperforms state-of-the-art algorithms such as PRM^∗^ and RRT^∗^, often by a significant margin. The speedups are particularly prominent in higher dimensions and in scenarios where collision checking is expensive, which is exactly the regime in which sampling-based algorithms excel. In this work, we utilize the advantages of FMT^∗^ algorithm to achieve the goal of motion planning and executing tasks.

#### 2.3.1. Assumptions

In designing the path planner using FMT^∗^ algorithm, we make the following two assumptions:

1. Decoupling between control and joint motion. The Raven-II robot uses cable-driven mechanisms, so the joint motion of the instrument arms are not only affected by one DC motor. The closer to the end-effector, the more complicated coupling motion would involve. In our ODE simulation environment, we do not model the cable coupling behavior, and we assume each actuator will control one joint motion only.
2. Fixed opening angle for the grasper. Each grasper consists of two jaws and, thus, has two DOFs. We can think of jaws as two independent DOFs or considering them as one part, and then it has one DOF as the center line of the grasper, another DOF would be the opening angle of the grasper, and these two representations are kinematically equivalent. In some cases, when the grasper is holding some object (e.g., holding the cube ring in our training scenario), we do want to keep the opening angle unchanged. So in our path planner, we only consider six DOFs, instead of seven.

#### 2.3.2. Problem Statement

As mentioned above, in this work, we consider motion planning problem for one arm, either left arm or right arm, because the case of dual arm planning problem can be treated as moving the arms one by one. This simplification will reduce the computation cost tremendously. The motion planning problem of Raven-II surgical robot in a surgical training environment can be stated as follows:

- *Inputs:* surgical environment Ψ and Raven-II robot *ℜ* described by mesh file objects, initial robot configuration *θ*_init_ for the moving arm (collision-free) when the path planning is enabled, goal robot configuration *θ*_goal_ for the same arm (collision-free) specified by the user, *n* number of collision-free configurations, and *r*_*n*_ connection radius
- *Output:* a feasible collision-free motion plan consisting waypoints Θ for one arm trajectory and each waypoint is a 6-dimensional vector including 6 joint positions.

Note that motion between two intermediate waypoints is dynamically feasible and free of collision. And if no path exists between start and end configurations, the algorithm terminates in finite time.

#### 2.3.3. FMT^∗^ Algorithm

We consider this planning problem for one robot arm. The planning objective is to find a dynamically feasible collision-free path while minimizing the overall cost function:

(1) $$\begin{array}{c} J\left(\sigma^{*}\right)=\min\left\{\eta_{1}\cdot c\left(\sigma \right)+\eta_{2}\cdot \int_{0}^{T}{\left|\alpha \left(t\right)\right|}^{2}dt,\;\sigma \;is\;feasible\right\}, \end{array}$$

in which *σ* is a feasible path in joint space, *c*(*σ*) is the arc length of *σ* with respect to Euclidean metric, *α*(*t*) is the end-effector's linear acceleration, and *η*_1_,  *η*_2_ are two user-defined coefficients to weight the effects of path length and velocity variations. The FMT^∗^ algorithm is outlined in Algorithm 1.

The description of the functions (e.g., SampleFree, Near, and Save) in the FMT^∗^ algorithm are described in [40]. In surgical planning, we should make sure that when connecting two waypoints on the path, no collision happens and the end-effector's velocity is smooth, that is, there is no jerky motion on the robot joints. So for evaluating the connectedness of two waypoints, rather than simply do linear or nonlinear interpolations as the kinematics level test, we integrate the robot dynamics with ODE and use it as the prediction to test whether by moving from one configuration to another as collision happens. This is the most expensive part of our implementation.

Similar to RRT^∗^ and PRM^∗^, FMT^∗^ also requires to explicitly specify radius when considering neighboring samples to achieve asymptotic optimality, which is given by the (3) in [40]. So we can write

(2) $$\begin{array}{c} r_{n}=\gamma {\left(\frac{\log\left(n\right)}{n}\right)}^{1/d}, \end{array}$$

for some positive *γ*. In our implementation, we normalized all joint positions in the range of [0, 1] to do uniform sampling. Then compute a conservative bound of *γ* using Monte Carlo simulation in order to find the *d*-dimensional Lebesgue measure of collision-free configurations with respect to all possible configurations.

Before trying to obtain collision-free samples, a decision-making module will determine which arm is supposed to move and call the corresponding FMT^∗^, because the two arms are slightly different in terms of transformations and kinematics chain [41].

Under this path planning with robot dynamics framework, joint controls at each time step can also be obtained as a byproduct of doing collision checking when trying to connect two samples. After a feasible path is computed, we can either apply the joint controls explicitly at each time step or through feedback control to execute the plan as a trajectory following problem. Since the first option is open loop, the error could accumulate over time. So, we will use PD controllers for each joint to track the desired trajectory in joint space when executing the motion plan autonomously in the simulator and send the joint positions to the Raven computer via internet if real robot motion is also needed to perform.

### 2.4. Spring-Damper Model for Haptic Force Feedback

In this section, we present the haptic force feedback mechanism. The use of haptic devices in teleoperated surgical robots has the potential of providing both cutaneous (tactile) and kinesthetic (force) information during exploration or manipulation of an object or environment. To the best of the authors' knowledge, even the latest commercial surgical system (da Vinci Xi) does not have haptic feedback feature. In robotic surgery, haptic feedback is useful in teleoperated palpation [42, 43]. Beyond this application, we expect that haptic feedback also can provide extra but crucial information to the operator about the status of the system when some uncertain events happen and before the errors are accumulated to some degree that the system is taken to an emergency stop. For human perception, our haptic rendering loop in the simulator also runs at 1000 Hz, otherwise, the user may perceive force discontinuities and a loss in fidelity [44].

We send the ROS published joint states in the Raven computer to the simulator through the network. From the physics engine (ODE) thread, we extract the joint velocities. We compute the end-effector velocities by using spatial manipulator Jacobian transformation:

(3) $$\begin{array}{c} \left[v\;\omega \right]T=J\overset{˙}{\theta }. \end{array}$$

The end-effector position is computed through the forward kinematics chain for both the robot and the model using the joint positions, as shown in

(4) $$\begin{array}{c} p=f\left(\theta \right), \end{array}$$

where *f* indicates the forward kinematics chain of the robot [41]. Then, the haptic force provided to the operator is given by

(5) $$\begin{array}{c} F=\left\{\begin{array}{c} K_{p}\left\|p_{model}-p_{robot}\right\|+K_{d}\left\|v_{model}-v_{robot}\right\|\\ 0.05,\;if\left\|p_{model}-p_{robot}\right\|>tol. \end{array}\right. \end{array}$$

And the force direction applied to the haptic device is given by

(6) $$\begin{array}{c} d=\frac{p_{model}-p_{robot}}{\left\|p_{model}-p_{robot}\right\|}. \end{array}$$

In this setup, if an adverse scenario happens, or the robot moves in an unexpected way, the haptic device will provide haptic cues to the operator. This provides awareness of impeding hazards, enabling the operator to take action or correct the robot behavior based on the internal model of the simulator.

### 2.5. Safety Hazard Injection

The Safety Hazard Injection engine in our simulator uses software-based fault injection techniques to recreate safety hazards observed during real surgical procedures. This enables evaluation of surgeon performance and response to safety hazards and prepares them for the best response actions to take in case of incidents.

Based on our preliminary review of almost 1500 accident reports on the da Vinci surgical system from the FDA MAUDE database, we identified three categories of common safety hazard scenarios as shown in Table 1. We simulate these scenarios by injecting faults into the Raven control software during the training scenarios. The possible causes of hazards may include accidental faults in robotic hardware or software, unintentional human operator errors, or intentional malicious attacks to the control system of the robot. For each safety hazard, Table 1 shows the potential accidental causes (column 3) and impact on patients and surgical team (column 5) based on representative examples from the real incidents reported to the FDA MAUDE database. The patient impacts represent clinical scenarios and response actions on which the robotic surgeons should be trained on.

The Safety Hazard Injection Engine consists of customized modules for (a) retrieving hazard scenarios, (b) generating software fault injection campaign and selecting fault injection strategy, (c) conducting fault injection experiments, and (d) logging and collecting data in an automated fashion [12]. The *Injection Controller* is responsible for starting, stopping, and automating the fault injection campaign. In a normal campaign execution, a Safety Hazard Scenario Library constructed based on the analysis of adverse events is accessed to retrieve the list of desired hazard scenarios. Then causal factors leading to each desired hazard scenario are simulated by selecting the fault injection parameters. Each hazard scenario includes a possible unsafe control action and a list of potential causal factors. An example unsafe control action would be a motor command is provided by the control software when there is a mismatch between the software state and hardware state of the robot. Faulty communication between software and hardware (e.g., through USB) is an example causal factor that might lead to such unsafe control action (see the third example in Table 1). Based on the causal factors involved in each hazard scenario, the analysis of Raven source code and software/hardware architecture, the *Fault Injection Strategies* module retrieves information on software functions which can most likely mimic the causal factors leading to the safety hazard as well as the key variables in those functions and their normal operating ranges. This information is translated to the parameters to be used by the fault injectors for simulating potential causal factors. The fault injection parameters include the location in the software function, the trigger or condition under which the fault should be injected, and the target variables to be modified by the injection (see column 4 of Table 1). Finally, the appropriate software-implemented *Fault Injectors* and the robot software are executed to conduct a fault injection experiment during a training scenario. At the end of each injection run, the injection parameters and data are collected for further analysis. For a more detailed description of Safety Hazard Injection Engine, refer to our previous work [12].

## 3. Results and Discussion

We present the experimental evaluations of the proposed hardware-in-the-loop simulator in this section. There are mainly two parts: (1) simulating safety hazards and their detection and (2) motion planning in a training environment with FRS model.

### 3.1. Fault-Free and Contact-Free Run for Model Verification

In this work, the robot dynamics is modeled for all 7 DOF, in ODE environment, compared to only 3 DOF were modeled in [45]. Our dynamic model is from joint torque to joint states, ignoring the motor dynamics and cable tensions. One reason for doing this is that the cable coupling introduces uncertainties in the model and nonuniformity of cable tension behaviors. In our experiment setting, we tighten the cables as tight as possible before the testing. Another reason is that the system is running at a 1000 Hz frequency, which gives very little margin for heavy computation and introducing even a small time delay that will cause system instability. Although the dynamics calculation is done on the Surgeon Console machine rather than the Raven computer, we still do not want to violate the timing constraints in each control loop.

**Figure 4:**
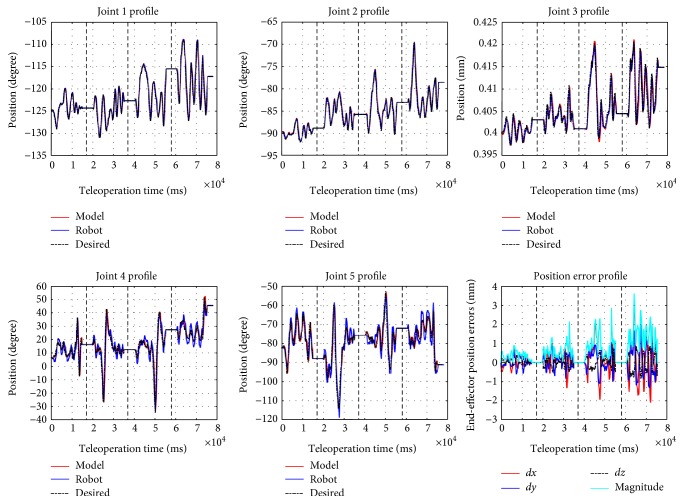
Comparison of the model and robot running data (up to 5 joints) and end-effector position error of (2.43 ± 1.72).

The joint torque vector *τ* is the controller output based on desired joint position obtained through inverse kinematics and current joint position. PD controllers are used for joints 1, 2, 4, 5, 6, and 7, and PID is used for joint 3, which is the tool insertion translational joint. A set of manually tuned PID gains make the system closely track the desired joint positions while keeping the joint torque/force *τ* within certain bounds. This means that the model is behaving like the robot rather than a system which has low damping and is fast enough to track reference signal.  Figure 4 shows the trajectories for arbitrary motions provided by the operator (black), the internal physics engine (dynamic model) calculations (red), and the real robot trajectory (blue) for the first five joints on the left robot arm (the right arm is identical to the left arm in terms of modeling and control). Through forward kinematics chain, one can obtain the end-effector position error.  Figure 4 shows the different portions of the trajectories (separated with dashed lines) corresponding to different teleoperation scaling factors, respectively, ranging from 0.05 to 0.2 with the spacing interval of 0.05. With larger motion scaling factors, the error also increases, because the modeling error for joints 4 and 5 are more sensitive to the scaling factor. These results verify the accuracy of the modeling in ODE environment, compared to the real robot trajectory.

### 3.2. Fault Injection to Robot Software

Many of the hazard scenarios shown in Table 1 may cause unexpected instrument movements and sudden jumps. In this section, we use the safety hazard injection engine to trigger the faulty commands at network layer and software hardware communication layer as in [8]. More specifically, to simulate the resulting safety hazard scenarios, we corrupt the motor commands sent to the robot hardware and the Omni commands sent to the Raven computer, as indicated by numbers in Figure 2.

In the experimental setup, we disabled the collision checking in ODE, that is, we did not consider the case where hard collision happens and will affect the robot dynamics too much. We inject periodic faults to (i) the first (shoulder) joint of the robot (which has stronger cable in the Raven-II) and (ii) the motion command data in network layer transmitting from the local machine to Raven computer, while the simulator receives the original “clean” Omni input. In the second case (in Figure 2), it is obvious that receiving the corrupted desired state data, the robot will follow the incorrect trajectory and end up deviating from the trajectory expected by the operator. From the fault-free run result as shown in Figure 4, we set the threshold of triggering the haptic force feedback when the end-effector positions between the model and the robot deviate more than 3 mm. In this section, we mainly focus on simulating and analyzing the resulting adverse events in the first case (i.e., injection of periodic faults to the motor commands).

#### 3.2.1. Simulation of Sudden Jump

In robotic surgery, many reported adverse events can be classified as unexpected joint motion in a small time interval, that is, sudden jump (the third scenario in Table 1). Although the causality can be many to one, we are able to reproduce this kind of adverse events and expose the surgeon during training phase by using our hardware-in-the-loop simulator incorporated with the safety hazard injection engine. We use haptic force feedback to provide information to the operator immediately, so that they can respond to the adverse events as quickly as possible, by emergency actions such as release the foot pedal to disengage the robot and triggering motor breaks (to avoid patient injuries). To simulate robot jump, during the teleoperation running mode, we inject constant motor command (can be zero or nonzero but within the valid range of motor's DAC command) to the shoulder joint at a specified time period. The underlying reason for the jump is the accumulation of position errors because the controller has to generate large torque commands to track the desired position once the robot goes back to the nominal run.

**Figure 5:**
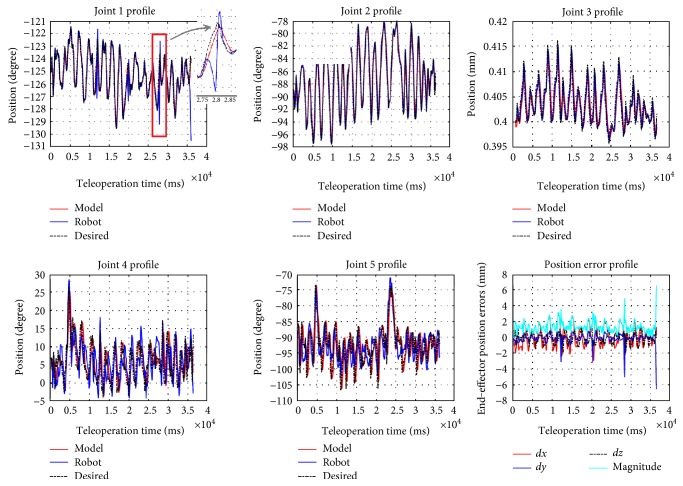
Robot and model trajectories during fault injection are enabled (with teleoperation scaling factor of 0.1).

Figure 5 shows the result of our hardware-in-the-loop simulator running with the fault injector. In this scenario, every 8 second after pedal down (teleoperation mode), the safety hazard injection engine corrupts the motor command sent to the shoulder joint and keeps the fault active for 300 cycles (300 ms). One can observe that the sudden jump behavior happened in joint 1 profile in Figure 5. The sudden jumps can happen many times (in this experiment, 4 times), while the operator may not notice them since the duration is quite short (a few milliseconds). Such abrupt jumps if only happen a few times during the procedure, they will leave no impression to the operator and he may even think that it is his own mistake. However, the sudden movements/jumps may happen due to hardware problems (see Table 1). The robot has the safety mechanisms to monitor the robot status and detect such faults, but in our fault injection experiments, we demonstrated the robot can jump frequently without triggering the robot's safety mechanisms [8, 12] (e.g., the robot stopped at the last jump due to the computed motor control is beyond the limit).

**Figure 6:**
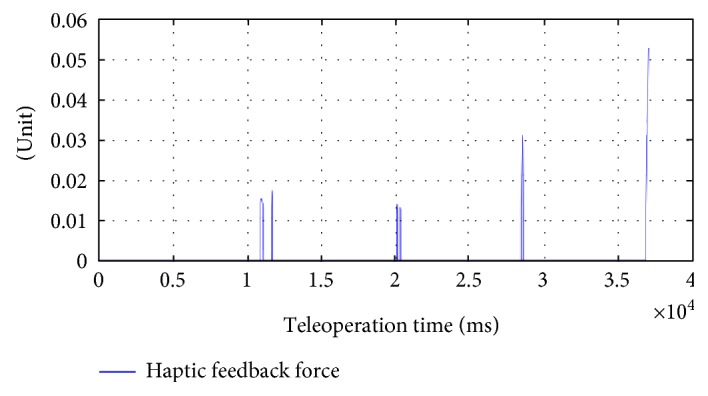
Haptic force feedback on the Omni device during fault injection.

Figure 6 shows the magnitude of the haptic force feedback provided to the Omni device using (5). The results show that we captured the adverse events exactly at the times the fault injection was performed and provided the feedback to the user in time. The haptic force is being saturated in a range that it will not interfere the normal teleoperation due to the passivity and high damping of the human operator. When a surgeon faces such a scenario in real surgery, possible mitigation strategies include slow down the motions or release the pedal to disengage the master and slave and call the technical help in the hospital (see the last column of Table 1).

### 3.3. Test Results on Path Planner

In this section, we evaluate the performance of the proposed Raven-II simulator with the integration of physics engine and the path planner. We performed all tests locally, that is, the simulator software and the Omni client software are running on the same Windows 7 machine, with Intel Core i7 CPU @ 3.50 GHz, to avoid the time delay caused by long distance communication over the network. And for all tests, the step size used in ODE is 0.001 second.

Initially, the robot arm is placed deeply inside the FRS training module. The test scenario is to achieve the motion sequences of retrieving the robot arm first from the surgical field and then moving forward to reach the cube ring. The opening area of the dome does not coincide with the pivot point (indicated by the yellow sphere in Figure 3) of the robot arm; thus, it is very easy to collide with the dome or loops. The user was asked to activate the planning mode, which means he can directly manipulate the arm to reach the target by any means with collision checking disabled. Once he arrived at the target configuration, the planner will record this configuration and start to run the planning algorithm. We evaluate the performance with respect to different objective functions, the number of collision-free samples, and the algorithm execution time.

**Figure 7:**
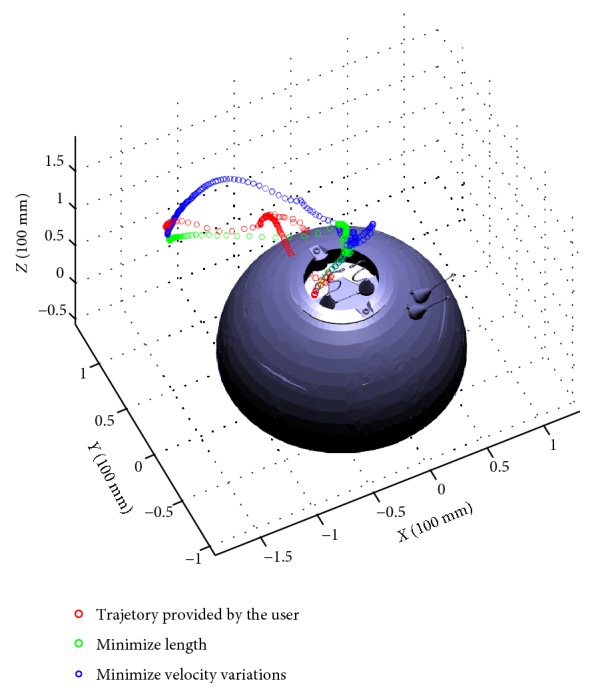
Three end-effector trajectories representing user's movement and two different optimization criteria.

Choosing 500 collision-free samples for the path planner, Figure 7 shows the end-effector (mass center of the two jaws) trajectories of user's movement when specifying the target configuration (red), minimizing 6-dimentional path length (green), and minimizing end-effector linear velocity variations (blue), respectively. They are the path tracking results rather than the waypoints for the planning results. We record the waypoints on every other loop time of ODE simulation loop so that they will be shown more clearly in the figure. The density of waypoints indicates the velocity. The sparser means the velocity is larger while more dense waypoints means the velocity is relatively slow. In our test scenario, it is difficult for the novice user to figure out a collision-free path to reach the cube ring's location while manipulating the robot arm. So in our interactive path planning setting, collision is allowed just simply for the user to quickly reach the target configuration. By choosing *η*_1_ = 1 and *η*_2_ = 0, we got a path with smaller length (planner returns 36 waypoints), while by choosing *η*_1_ = 0 and *η*_2_ = 1, we got a path with smaller velocity variations (planner returns 54 waypoints).

**Figure 8:**
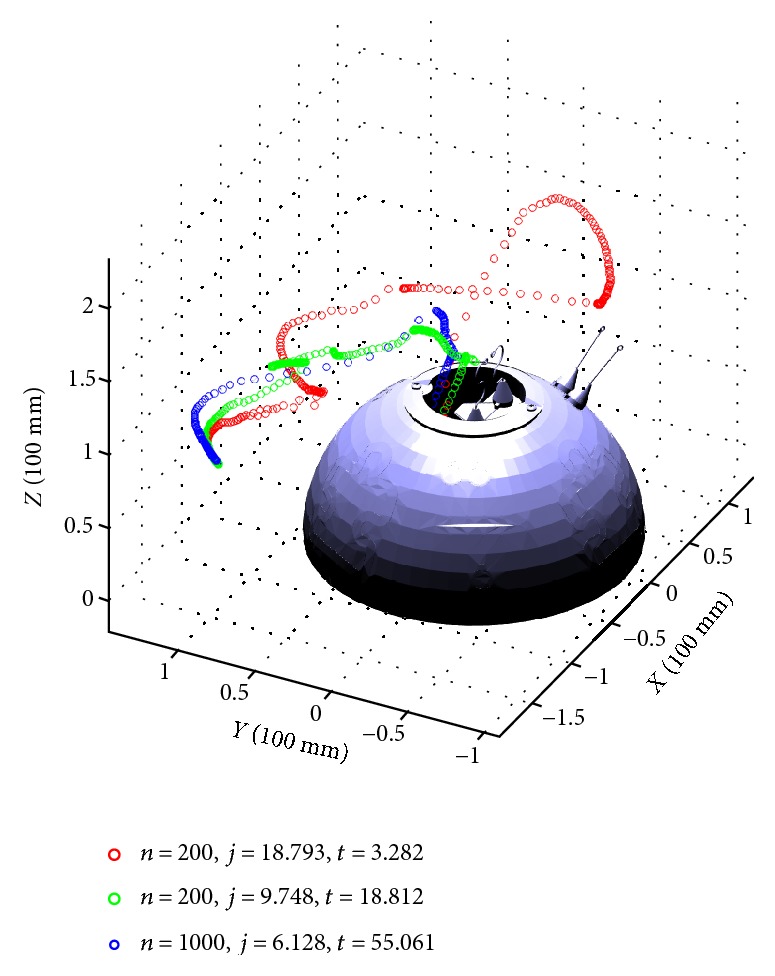
Three end-effector trajectories representing returned by FMT^∗^ using different number of samples.

Next, we compare the planning performance of using a different number of collision-free samples and verify that the algorithm can converge to the optimum with respect to the cost function. In this test scenario, we choose *η*_1_ = 0.1 and *η*_2_ = 0.5. As shown in Figure 8, we observe that as the number of samples increases, the cost becomes smaller and the algorithm running time becomes longer due to heavier computation in the “for” loop of the algorithm.

Table 2 shows the algorithm performance with *η*_1_ = 0.1 and *η*_2_ = 0.5 and autonomous tracking performance. Each test scenario runs the planning algorithm and autonomous tracking 20 times to get the average result. In Table 2, “returned reference waypoints” means the number of waypoints returned by FMT^∗^, and going from one position to another is dynamically feasible in the planning phase. However, as the number of samples increases, the path gets shorter, and thus in path tracking, it would be closer to the obstacle (wall of the dome) and result in lower success rate (more chances to collide). One way to resolve this issue is to include an additional term in cost function as maximizing the obstacle clearance distance. In this training setup, the planning time and the execution time are acceptable if using appropriate number of samples to start the algorithm.

## 4. Conclusions

We have demonstrated a general framework for robot-assisted surgical simulators for a more robust and resilient robotic surgery. With the goal of providing high-fidelity surgical training in simulation, we created a hardware-in-the-loop simulator platform. We integrate the simulator with a physics engine and a state-of-the-art path planning algorithm to help surgeon acquire an optimal sense of manipulating the robot instrumental arm and achieve autonomous motion of the surgical robot. We integrated a safety hazard injection engine integrated with the simulator software to reproduce safety hazards happened in real surgery. A haptic force feedback mechanism was designed to provide surgeon an extra modality of information about the robot status when unexpected motion happens. Delivering the safety alarm to the surgeon by haptics is an efficient way of capturing such occurrences but will need additional human factor studies.

The future work includes providing haptic feedback to guide the operator moving along the preplanned optimal path to perform training tasks, for example, use of gripper to grasp a ring. Since the current teleoperation mechanism uses incremental motion of the master and maps to the end-effector of the slave robot to resolve the two different workspaces of master and slave (thus using the pedal to disengage/engage and reconfigure the master), we can provide visual or audio cues to the operator once it approaches the workspace boundary of the haptic device. With haptic force guidance, we believe we can further reduce the need for supervisory by the expert surgeon during training.

## Figures and Tables

**Algorithm 1:**
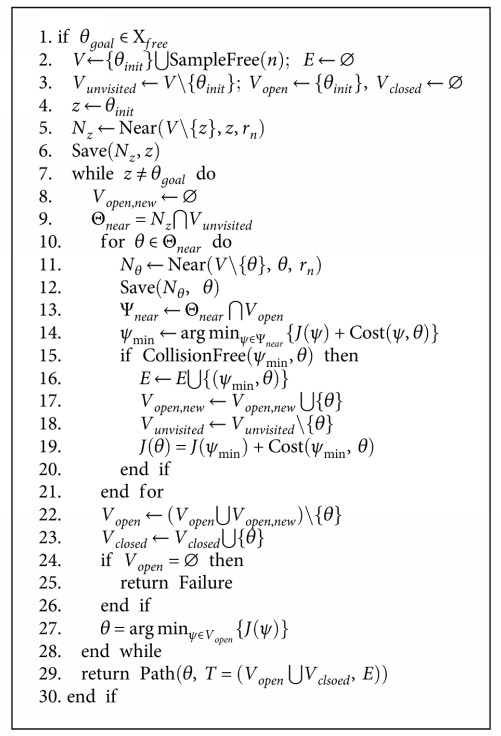
FMT^∗^.

**Table 1:** Three common safety hazard scenarios, with corresponding examples from real incidents reported to the FDA MAUDE database.

Safety hazard scenario (outcome)	Unsafe control action example	Possible causal factors (accidental failures)	Raven-II simulation		Impact (clinical scenarios for safety training) [example]
			Target software module	Target variables	
System temporarily unavailable *(recoverable system error)*	A user command is provided but not followed by the robot.	Improper operator actions or console control malfunctions	Network-layer thread (network_layer)	User-desired	Restart the system [MAUDE 3293519] Troubleshoot error contact manufacturer
				(i) Position	
				(ii) Orientation	
				(iii) Grasper angle	
				(iv) Foot pedal	

System permanently unavailable *(nonrecoverable system error)*	A motor command is provided by the robot control, but it is not followed by the motors.	Sensor (encoder) failure	Control thread (get_USB_packet)	USB board	Convert the procedure [MAUDE 2663924] Reschedule [MAUDE 3275500]
				(i) Address	
				(ii) Returned status	
				USB board	Report to manufacturer
				(i) Address	
				(ii) Returned status	

Unintended movement of robotic arms *(sudden jump)*	A command is provided by the robot control to motors while the calculated next position is at large distance (big jump) from current position.	Actuator failures	Control thread (put_USB_packet)	Commands to robot joints	Puncture of artery [MAUDE 1590517] Bleeding of uterine tube [MAUDE 2120175]

**Table 2:** Performance evaluation result.

Sample number	Planning time (s)	Path tracking time (s)	Returned reference waypoints number	Cost	Success rate (%)
200	3.676	4.705	13.5	21.9	90
500	15.935	4.277	14.3	11.1	85
1000	47.708	3.909	11.1	6.5	85
2000	216.373	3.688	11.1	5.7	75
